# Graph Theory on Brain Cortical Sources in Parkinson’s Disease: The Analysis of ‘Small World’ Organization from EEG

**DOI:** 10.3390/s21217266

**Published:** 2021-10-31

**Authors:** Fabrizio Vecchio, Chiara Pappalettera, Francesca Miraglia, Francesca Alù, Alessandro Orticoni, Elda Judica, Maria Cotelli, Francesca Pistoia, Paolo Maria Rossini

**Affiliations:** 1 Brain Connectivity Laboratory, Department of Neuroscience and Neurorehabilitation, IRCCS San Raffaele Roma, Via Val Cannuta, 247, 00166 Roma, Italy; fabrizio.vecchio@uniecampus.it (F.V.); pappaletterachiara@gmail.com (C.P.); alufrancesca@yahoo.it (F.A.); alessandro.orticoni@gmail.com (A.O.); paolomaria.rossini@sanraffaele.it (P.M.R.); 2 Department of Theoretical and Applied Sciences, eCampus University, 22060 Novedrate, Italy; 3 Department of Neurorehabilitation Sciences, Casa Cura Policlinico, 20144 Milano, Italy; e.judica@ccppdezza.it; 4 Neuropsychology Unit, IRCCS Istituto Centro San Giovanni di DioFatebenefratelli, 25125 Brescia, Italy; mcotelli@fatebenefratelli.eu; 5 Neurological Institute, Department of Biotechnological and Applied Clinical Sciences, University of L’Aquila, 67100 L’Aquila, Italy; francesca.pistoia@univaq.it

**Keywords:** EEG, graph theory, functional connectivity, eLORETA, Parkinson’s disease, small world

## Abstract

Parkinson’s disease (PD) is the second most common neurodegenerative disease in the elderly population. Similarly to other neurodegenerative diseases, the early diagnosis of PD is quite difficult. The current pilot study aimed to explore the differences in brain connectivity between PD and NOrmal eLDerly (Nold) subjects to evaluate whether connectivity analysis may speed up and support early diagnosis. A total of 26 resting state EEGs were analyzed from 13 PD patients and 13 age-matched Nold subjects, applying to cortical reconstructions the graph theory analyses, a mathematical representation of brain architecture. Results showed that PD patients presented a more ordered structure at slow-frequency EEG rhythms (lower value of SW) than Nold subjects, particularly in the theta band, whereas in the high-frequency alpha, PD patients presented more random organization (higher SW) than Nold subjects. The current results suggest that PD could globally modulate the cortical connectivity of the brain, modifying the functional network organization and resulting in motor and non-motor signs. Future studies could validate whether such an approach, based on a low-cost and non-invasive technique, could be useful for early diagnosis, for the follow-up of PD progression, as well as for evaluating pharmacological and neurorehabilitation treatments.

## 1. Introduction

Parkinson’s disease (PD) is a slowly progressive movement condition and one of the most common neurodegenerative disorders, affecting worldwide approximately 1–2% individuals older than 60 years old. Although it is well established that α-Synuclein, linked genetically and neuropathologically to PD, is one of the main components of Lewy body deposits in the substantia nigra, leading to dopaminergic dysfunction in the basal ganglia, the diagnosis of PD is still based on the clinical history and the physical examination of the patient [1,2]. Indeed, nowadays, there is no standard diagnostic tool for PD detection, particularly in the early stages of the disease, except for the DAT-SCAN, a relatively expensive and invasive neuroimaging technique utilizing a radioligand [3]. The clinical manifestations of PD are predominantly characterized by motor symptoms, such as bradykinesia, resting tremor, gait disturbance, rigidity and postural instability [4,5,6]. Moreover, PD is classically distinguished into two motor subtypes, tremor-dominant (TD) and non-tremor dominant (nTD), the latter mainly characterized by postural instability, gait difficulty and akinetic–rigid syndrome [7,8]. Furthermore, most PD patients may suffer from non-motor-symptoms such as autonomic dysfunction, hyposmia, sleep disorders, cognitive impairments or psychiatric disturbances [9,10]. These symptoms lead to serious disability and poor quality of life among patients. Usually, the presence of both cardinal motor dysfunction (i.e., akinesia, rigidity, postural instability and resting tremor) and response to levodopa supports the diagnosis of PD [3,11,12,13].

It may be challenging to distinguish typical PD features from signs of normal aging, as well as from manifestations of atypical parkinsonism [14]. In fact, PD motor symptoms may be mistaken for motor impairments resulting from normal aging, i.e., age-related parkinsonism [4,5,6,14,15,16]. The dysfunction of the cortico–striatal–thalamic–cortical loops seems to lead to the hallmark motor features of PD, including tremor, bradykinesia and rigor [17,18]. Although the exact mechanism underlying the pathophysiology of PD is unknown, increasing evidence has suggested that it could be associated with abnormal cortical–subcortical–cortical connectivity network organization involving a widespread group of brain regions orchestrated by the basal ganglia [19,20]. Indeed, PD impacts subcortical pathways, leading to dysfunctional automatic movement control, which has been suggested to be followed by a compensatory shift to an enhanced voluntary cortical control [21,22,23]. This degenerative disorder tends to become a serious social burden; therefore, new approaches to improve PD early detection are required in order to uncover sensitive and reliable biomarkers and to implement efficient treatment at the appropriate time.

In recent years, brain dynamics in PD have been studied with magnetic resonance imaging (MRI), which has been useful in identifying structural lesions associated with other forms of parkinsonism, vascular pathologies or neoplasms, and to measure the degree and the distribution of brain atrophy [24]. In particular, resting-state functional magnetic resonance imaging (rs-fMRI) has investigated functional connectivity of the motor network in PD during rest, showing significantly decreased functional connectivity in the supplementary motor area, left dorso-lateral prefrontal cortex and left putamen in patients as compared to healthy controls [25,26]. Despite significant evidence for the relevance of neuroimaging in assessing parkinsonian patients, none of the currently available neuroimaging techniques are specifically recommended for routine use in daily clinical practice for PD [24]. A potentially useful diagnostic tool for evaluating PD patients could be electroencephalography (EEG) [27], a non-invasive technique able to describe the brain electric activity with the benefit of high temporal resolution: as compared to fMRI, EEG could have similar diagnostic accuracy but significantly lower costs [28,29]. Many studies on the spectral analysis of EEG data have identified numerous pathological brain rhythm alterations in PD patients; indeed, it was shown that the motor symptoms in PD patients were related to increased activity in the alpha band [30,31]. Resting EEG data analyses showed a decrease in beta power [32,33] and a remarkable increase in theta and low alpha powers in PD patients in comparison to controls [34,35]. Furthermore, a number of studies concluded that the most common abnormality of EEG in PD patients is the generalized slowing of brain activity. For instance, Serizawa and colleagues [36] showed that PD patients exhibit a diffuse slowing in quantitative EEG in comparison to normal controls; Bosboom and coworkers [37] found that PD is characterized by a slowing of resting-state brain activity involving the theta, beta and gamma bands; Stoffers and others [34] confirmed that the slowing of oscillatory brain activity is a stable characteristic of PD without dementia.

Another approach used to investigate the brain dynamics and to characterize the structure and dynamics of relevant networks is graph theory, a mathematical representation of the brain architecture. In fact, some studies have shown that PD is characterized by alteration of the loops of cortical–subcortical pathways [21,22]. Thus, an approach based on EEG brain connectivity architecture analysis could be very helpful in identifying the specific features of PD patients. Previous evidence has shown how to apply graph theory to the diagnosis of Alzheimer’s disease [38] and also to the evaluation of vascular dementia [39], schizophrenia [40] and depression [41]. A recent study investigated the resting-state brain network topology in PD patients in relation to clinical scales of disease progression using magnetoencephalography and concepts from graph theory [42]. In particular, the results indicated that changes in graph theory parameters are very early promising markers of PD progression, associated with the deterioration of motor function and cognitive performance. Utianski and colleagues [43] revealed that network measure alterations are visible in PD patients, suggesting an abnormal interaction between cortical areas. This contributes to PD symptoms, demonstrating how graph theory analysis by EEG is a robust form of analysis for various stages of PD. Despite this bulk of evidence, graph theory has been poorly applied to the study of brain dynamics in PD patients; in particular, to the best of our knowledge, no study has previously investigated the parameter of the small world (SW) organization applied to cortical sources.

Keeping in mind the above evidence, the aim of the present pilot study was to explore the differences in resting-state brain connectivity between PD patients and NOrmal eLDerly (Nold) subjects applying graph theory—in particular, SW analysis—to cortical sources.

## 2. Materials and Methods

### 2.1. Participants

Thirteen patients diagnosed with PD and thirteen Nold subjects, harmonized for sex (8 female) and age (61.54 ± 2.47, mean ± standard error), were recruited in the study groups. The entire experiment was started after receiving the informed consent of each participant, according to Code of Ethics of the World Medical Association (1997), and all the procedures met the requirements of the Declaration of Helsinki. The EEG recording was performed in accordance with safety guidelines. The diagnosis of PD was based on the medical history, neurological and physical examinations, as well as on the response to levodopa drugs. The exclusion criteria included atypical parkinsonism, use of neuroleptic treatments, antidepressants and dopamine blocking drugs, alcohol abuse, the presence of other neurological or psychiatric conditions and any other severe illness. In the Nold group, the subjects were healthy, without symptoms or history of neurological or psychiatric disorders.

### 2.2. Data Recordings and Preprocessing

Resting-state eyes-closed conditions were recorded from EEG for at least 6 min. During the recordings, participants were placed on a comfortable armchair. The EEG time series were recorded through 19 electrodes (Fp2, F4, C4, P4, O2, F8, T4, T6, Fp1, P3, C3, P3, O1, F7, T3, T5, Fz, Cz, Pz) positioned with a montage resulting from the International 10–20 scheme.

Vertical and horizontal electrooculography channels (EOGs) were positioned to check eye blinking artifacts. The impedance of all electrodes was kept below 5 KΩ. The data (sampling rate frequency of 256 Hz) were analyzed in Matlab (MathWorks, Natick, MA, USA) using functions built from EEGLAB toolbox (Swartz Center for Computational Neurosciences, La Jolla, CA, USA) [44,45,46].

The EEG data were collected with a band-pass finite impulse response (FIR) filter from 0.2 to 47 Hz. Then, they were segmented in 2 s duration epochs and main artifacts in the EEG signal (i.e., eye movements, scalp muscle contraction and cardiac activity) were removed by an EEG expert and by Infomax ICA algorithm [47,48], which allowed the separation of independent component sources of the multichannel EEG recordings [49,50,51,52,53], as implemented in the EEGLAB toolbox. The artifact removal procedure was realized keeping at least 5 min for each subjects.

### 2.3. Functional Connectivity of Cortical Sources Analysis

By means of the software of exact Low Resolution Electromagnetic Tomography (eLORETA) [54], brain connectivity was calculated on Regions of Interest (ROIs) according to the Brodmann areas (Bas): 42 ROIs for each hemisphere (left and right) (BAs: 1, 2, 3, 4, 5, 6, 7, 8, 9, 10, 11, 13, 17, 18, 19, 20, 21, 22, 23, 24, 25, 27, 28, 29, 30, 31, 32, 33, 34, 35, 36, 37, 38, 39, 40, 41, 42, 43, 44, 45, 46, 47). The ROI were used to compute the brain functional connectivity from the estimation of its electric neuronal activity. In particular, the intracortical Lagged Linear Connectivity, namely current density time series, was extracted between all possible pairs of the 84 ROIs using the algorithm of “all nearest voxel” [54,55]. For each subject, the current density estimation was calculated for seven independent EEG frequency bands, namely delta (2–4 Hz), theta (4–8 Hz), alpha 1 (8–10.5 Hz), alpha 2 (10.5–13 Hz), beta 1 (13–20 Hz), beta 2 (20–30 Hz) and gamma (30–45 Hz) [56].

### 2.4. Graph Analysis

**Figure 1:**
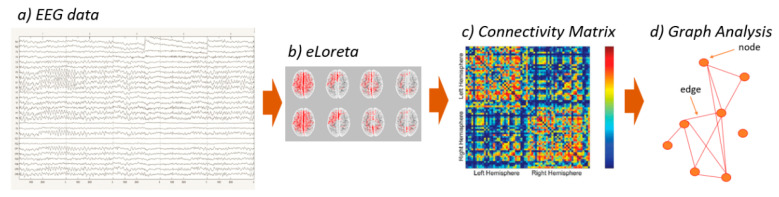
EEG analysis pipeline from the raw data to the computation of small world parameter. (**a**) The first step is the analysis of EEG raw data to remove artifacts; (**b**) the clean data were used to estimate the electric neuronal activity of the ROIs; (**c**) intracortical Lagged Linear Connectivity was extracted in a connectivity matrix between all possible pairs of the 84 ROIs and (**d**) it was used to weight the link of the networks’ edges.

A network is a mathematical representation of a complex system. In the last decade, in several studies, the brain was defined by a set of nodes and links, where the first usually represent brain regions while the second ones represent the functional connections between nodes. A weighted graph is a mathematical structure of vertices that may be linked to each other by different and variable weights. In the current study, the values of connectivity computed between all pairs of ROIs for each frequency band and for each subject were used as the weight of the graph edges in the following graph analyses (the EEG analysis pipeline is reported in Figure 1). The nodes were defined as the ROIs, and the links of the network were weighted by the Lagged Linear Connectivity values [57]. The small world (SW) index was defined after the calculation of characteristic path length and clustering coefficient, which represent, respectively, global connectedness and local interconnectedness [58]. SW was calculated as the ratio of the normalized clustering coefficient and normalized path length (obtained by dividing the values previously computed by the values obtained by the mean of each parameter in all the frequency), and it is used to describe a balance between segregation and integration [49,59].

### 2.5. Statistical Evaluation

Data comparisons were analyzed by the statistical analysis of variance (ANOVA) design for the SW index between the factors Group (PD, Nold) and Band (delta, theta, alpha 1, alpha 2, beta 1, beta 2, gamma), after the evaluation of the normality of the data using the Kolmogorov–Smirnov test, confirming that the hypothesis of Gaussianity could not be rejected. Data were also corrected by the Greenhouse and Geisser correction for protection against a possible violation of the sphericity assumption in the repeated-measures ANOVA. In addition, the post-hoc Duncan’s test with a significance level at 0.05 was performed.

## 3. Results

**Figure 2:**
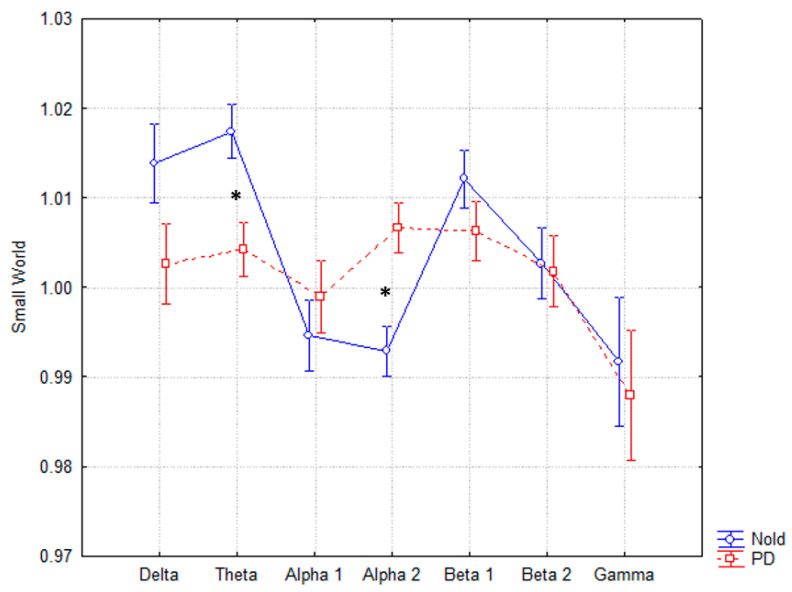
Small world trend in the two groups of subjects. Statistically significant interactions (F(6, 144) = 2.1213, *p* < 0.05) between SW, Group (PD, Nold) and Band (delta, theta, alpha 1, alpha 2, beta 1, beta 2, gamma) are reported. In particular, the post-hoc test showed statistical differences in the theta and alpha band. * Statistically significant with a significance level of 0.05.

The ANOVA for the evaluation of the SW index showed a statistically significant interaction (F(6, 144) = 2.1213, *p* < 0.05) between both factors, Group (PD, Nold) and Band (delta, theta, alpha 1, alpha 2, beta 1, beta 2, gamma), as reported in Figure 2. The post-hoc Duncan’s test showed statistical differences in theta (*p* < 0.05) and alpha 2 (*p* < 0.05). In particular, the SW index in Parkinson’s showed lower values (more structured network) in theta and higher (less organized network) in alpha 2 compared to controls. All values (mean, standard error and *p* values for each band) are reported in Table 1. 

## 4. Discussion

Parkinson’s disease is a neurodegenerative disorder characterized by typical motor as well as non-motor symptoms. The motor symptoms include bradykinesia, muscular rigidity, rest tremor and postural impairment. Non-motor impairments include hyposmia, sleep disorders, cognitive impairment, psychiatric symptoms and autonomic dysfunction [4,60]. As PD is caused by the prominent death of dopaminergic neurons in the substantia nigra pars compacta, and several neuroimaging studies suggest that striatal dopamine reduction causes disorders affecting brain circuits governed and orchestrated by the basal ganglia, various mathematical approaches have been applied in order to identify and visualize abnormal connectivity in brain networks in PD [61,62,63,64]. Among these, network science and graph theory through EEG data have been widely used to investigate the organization of human brain networks, simplifying the brain as a graph composed of nodes (representing regions) and edges (representing functional connectivity among the nodes) [65,66,67,68]. In fact, it has been demonstrated that graph theory is able to evaluate the dynamic consequences in large cortical networks [69] and can significantly contribute to explaining neurological brain function and dysfunction [38,63]. Within this theoretical framework, the present pilot study aimed to investigate the functional brain connectivity differences between PD and age-matched healthy subjects—in particular, by means of small world (SW) network analysis in closed-eyes resting-state EEG recordings. The main goal of the current study was to demonstrate that the processes of cerebral integration and segregation undergo variations in the resting-state EEG, revealing changes in the brain during pathological conditions as compared to physiological ones.

The results showed that PD patients presented a more ordered low-frequency EEG rhythm structure (lower value of SW) than age-matched healthy subjects, particularly in the theta band (4–8 Hz). Conversely, in the high-frequency alpha band (10.5–13 Hz), PD patients presented more random organization (higher value of SW) than age-matched healthy subjects (Table 1, Figure 2). 

The observed result of SW reduction in the theta frequency band in the PD group could be interpreted both as a loss of efficiency of the network communication flow among brain regions [65,70] and as a reflection of the abnormal motor activity. In fact, the tremor—a typical symptom of PD—is associated with neuronal oscillations in the ventral intermediate (Vim) nucleus of the thalamus and in the subthalamic nucleus (STN) circuit exactly in the theta frequencies (4–7 Hz) [71,72]. Several studies indicate that atypical neuronal activity of the STN plays a pivotal role in the pathophysiology of parkinsonian motor symptoms; indeed, either lesioning or deep brain stimulation (DBS) of the Vim and STN, respectively, significantly reduce tremors [73,74]. Furthermore, previous studies have demonstrated that EEG spectral analysis in the resting state in PD patients increased in slower- and decreased in faster-frequency bands, suggesting a slowing of PD patients’ cortical activity [29,37,75]. 

Others [76] have revealed that the EEG cortical sources in the theta frequencies are associated with a pathological synchronization of the brain motor systems related to tremor or sensorimotor integration. Moreover, a magnetoencephalography (MEG) study—in which the authors computed the coherence between tremor and several oscillatory rhythms [77]—has revealed that the tremor diffusely influences the MEG signal, modifying the power, especially in the theta band. 

For the higher-frequency bands, the SW increase in the alpha 2 band in PD patients could be interpreted as a possible biomarker of a cognitive decline in the early phase of PD. High alpha rhythm (10.5–13 Hz) reflects the physiological modalities of the thalamo–cortical and cortico–cortical loops, which facilitate and inhibit the transmission of impulses and the processing of sensorimotor information flow [78,79,80,81]. 

Several studies have demonstrated that a decrease in alpha power is correlated with reduced brain region synchronization and integration, namely a more randomized network, which reflects cognitive dysfunction [43,82,83]. In general, the alpha band constitutes an important characteristic of normal EEG activity at rest; a disruption of these rhythms might be interpreted as an EEG marker of altered cortical functioning and impaired information processing. Vecchio and colleagues [84] have revealed that an increase in the alpha SW parameter, derived from EEG data, can distinguish between a neurodegenerative status, as Alzheimer’s disease, and a healthy elderly brain condition. In fact, they observed that the SW index in the alpha band increased in the pathological condition rather than the physiological one. In other studies [2,85], the EEG recordings of PD patients were analyzed through several indexes of graph theory, demonstrating evidence of network breakdown that correlates with decreased cognitive performance. 

As motor impairments are particularly relevant in PD, we made further considerations towards the alpha 2 band. Indeed, numerous studies have revealed that alpha and beta band-related networks may be linked to attentional deficits and motor impairments. In fact, a correlation between alpha and movement organization—namely a PD motor and rigidity subscale—was found [86,87]. In the current study, an increase in terms of SW in alpha 2, which means more random network organization, might be an early sign of motor dysfunction in PD patients. In line with our results and hypothesis, Olde Dubbelink and collaborators [42] showed a decrease in the path length parameter in the alpha 2 band in PD patients compared to healthy subjects, which could be associated with a more random network organization. 

Therefore, according to our findings, a lower SW value in the theta band and a higher value in the alpha band represent functional disconnections that could be interpreted as biomarkers of motor impairments typical of PD and a reduction in the performance of cortical networks. 

In conclusion, as Parkinson’s disease represents a leading public health challenge in the older population, an early diagnosis stage has become an important goal of the current clinical pharmacological and rehabilitation treatments. To this aim, it appears that graph theory applied to EEG data, a relatively simple, non-invasive, low-cost and widely available diagnostic tool, has proved very useful in identifying differences in brain network behaviors in subjects with PD-related symptoms. Future studies will indicate whether graph theory applied to EEG analysis actually represents an innovative biomarker to support PD diagnosis in the early stages and to define the pharmacological and rehabilitation strategy that is most suitable for the cognitive and functional recovery of patients with Parkinson’s disease. Further studies may investigate the EEG connectivity patterns, through graph theory, in different populations of patients with PD. This may contribute to understanding whether different PD forms, such as tremor-dominant and non-tremor dominant, also differ in brain network behaviors as a prerequisite for the determination of severity and progression.

## 5. Conclusions

The results of the present pilot study show that resting brain networks exhibit a different “small world” organization between Parkinson’s patients and control subjects. The results suggest that Parkinson’s disease globally modulates the cortical connectivity of the brain, modifying the underlying functional organization, and that this modulation could be linked to changes in the synaptic efficiency of the motor network and related areas of the brain. Future studies could verify whether the SW modulations are also observable in younger subjects compared to elderly ones or pathological patients. Evaluating this parameter could be helpful for the early diagnosis and treatment of PD, for following the progression of the disease and for planning neurorehabilitation treatments.

## Figures and Tables

**Table 1:** Small world values in the two groups of subjects described by means of mean ± standard error (SE). Duncan’s post-hoc test confirmed the statistical differences in the theta (*p* < 0.05) and alpha 2 (*p* < 0.05) bands. NS = not significant.

	Delta	Theta	Alpha 1	Alpha 2	Beta 1	Beta 2	Gamma
PD							
Mean	1.002604	1.004257	0.998959	1.006675	1.006256	1.001777	0.987933
SE	0.005757	0.00366	0.003947	0.002724	0.003999	0.003196	0.007649
Nold							
Mean	1.013802	1.017418	0.994609	0.992857	1.012101	1.002637	0.991665
SE	0.002409	0.002255	0.004055	0.002825	0.002271	0.004642	0.006738
*p* value	NS	*p* < 0.05	NS	*p* < 0.05	NS	NS	NS

## Data Availability

The data that support the findings of this study are available on request from the corresponding author.
